# Analyzing retraction trends in urology: a comprehensive study over the last decade

**DOI:** 10.1007/s00345-025-05764-5

**Published:** 2025-06-25

**Authors:** Julio Yanes, Daniel Ajabshir, Aravindh Rathinam, Archan Khandekar, Jonathan Katz, Robert Marcovich, Hemendra N. Shah

**Affiliations:** 1 Leonard M. Miller School of Medicine, University of Miami, Desai Sethi Urology Institute, 1120 NW 14th St #2107, 15th Floor, Miami, FL 33136 USA; 2 Herbert Wertheim College of Medicine, Florida International University, 11200 SW 8th St, Miami, FL 33199 USA

**Keywords:** Retraction trends, Urology, Research integrity, Open-access publishing, Urological literature, Scientific misconduct

## Abstract

**Objective:**

To investigate why retractions in academic literature have risen substantially, leading to rising concerns about research reliability and integrity. While retraction trends have been explored across disciplines, urology-specific factors remain underexamined. This study investigates 292 retracted urological publications from 2014 to 2024, focusing on open-access journals to analyze how publishing models influence retraction trends.

**Methods:**

A retrospective analysis of retracted urological publications was conducted using the PubMed database. The study employed 84 MeSH search terms to identify articles and categorize them by research type, journal impact factor, citation count, geographical distribution, and retraction reasons. Statistical analyses were performed to assess associations between retraction characteristics.

**Results:**

The most common reason for retraction (90.4%) was discrepancies in data availability or research description, with systematic publication manipulation accounting for 5.1%. The majority of retractions (84.5%) originated from China. Journals with higher impact factors exhibited longer recall times for retractions but no significant difference in citation count at recall.

**Conclusion:**

This study highlights the increasing frequency of retractions in urology and identifies key factors influencing these trends. Geographic disparities, open-access models, and journal impact factors play significant roles. Addressing research integrity requires improved editorial oversight, standardized reporting guidelines, and enhanced detection of publication misconduct.

## Introduction

Retractions in scientific literature are not a new phenomenon, with the first recorded case in PubMed dating back to 1977 for a 1973 paper in Nature [1]. Since then, retractions in academic literature have significantly increased, raising concerns about research reliability. Between 2004 and 2013, the number of retracted articles grew tenfold [2], and subsequent studies show a continued rise across disciplines. Retractions occur due to methodological flaws, data inaccuracies, or ethical violations like plagiarism or falsification [3, 4]. While earlier retractions were often due to unintentional errors, the recent retractions are more due to misconduct, including data fabrication [5]. Understanding these trends is crucial, given their direct impact on clinical decision-making and patient care.

In urology, the first retraction occurred in June 2000, with an article entitled Prostate Cancer Screening in the Tyrol Austria: experience and results in the European *Journal of Cancer.* This event highlighted the field’s growing awareness of research integrity. As of now, only two key studies have examined retractions in urology: that of Mena et al. (2019), which analyzed retractions from 1999 to 2018, focusing exclusively on subscription-based journals and original research articles, and that of Alvermann et al. (2023), which reviewed 78 retractions from 2000 to 2020, comparing urology with other surgical fields but lacking a detailed focus on urology-specific trends [3, 6]. Neither study analyzed in detail the impact of publishing models (open-access vs. subscription-based) on retraction trends. Our study addresses this gap by using an expanded search strategy with 84 MeSH terms to capture a broader range of retracted articles, covering retractions from 2014 to 2024 for a more up-to-date evaluation of urological research integrity. Additionally, we examine recall time in relation to journal impact factor, geographic shifts in retractions, citation patterns, and retraction reasons beyond misconduct, as well as discrepancies across journal types and publishing houses, providing a more comprehensive assessment of research integrity in urology.

By identifying key drivers of retractions, we aim to provide readers with a comprehensive overview of the current status of retractions in urological literature. This information may assist urological researchers in taking necessary precautions to prevent retractions in the future.

## Materials and methods

### Study design

We performed a retrospective review of Urology literature for retracted articles using a MeSH search and retracted articles filter in PubMed. This study was exempt from our institutional IRB as patients were not included in this research.

## Methods

We performed a retrospective analysis of the PubMed database to identify retracted urological manuscripts published between 2014 and 2024. A total of 84 keywords were used, such as “Retracted Publication”, “Prostatic Hyperplasia”, “Prostatitis”, “Cystitis”, “Urinary Incontinence”, “Cystectomy”, “Cystoscopy”, “Enuresis”, “Erectile Dysfunction”, “Lithotripsy”, “Hematuria”, “Testicular Hydrocele”, “Hydronephrosis”, “Hypospadias”, “Kidney Calculi”, “Nephrectomy”, “Urinary Bladder Neurogenic”, “Orchiectomy”, “Penile Induration”, “Priapism”, “Kidney”, “Prostate”, “Prostatectomy”, “Transurethral Resection of Prostate”, “Suburethral Slings”, “Cystocele”, “Seminal Vesicles”, “Ureter”, “Urethra”, “Ureterocele”, “Urodynamics”, “Urolithiasis”, “Varicocele”, “Vas Deferens”, “Vasectomy”, “Vasovasostomy”, “Vesicovaginal Fistula”, “Ureteroscopy”, “Adrenal Glands”, “Adrenal Gland Neoplasms”, “Adrenalectomy”, “Adrenergic alpha-1 Receptor Antagonists”, “Kidney Neoplasms”, “Androgen Antagonists”, “Testosterone”, “Renal Artery”, “Kidney Transplantation”, “Balanitis”, “Urethral Stricture”, “Bladder Exstrophy”, “Epispadias”, “Transurethral Resection of Bladder”, “Epididymitis”, “Circumcision Male”, “Penile Neoplasms”, “Testicular Neoplasms”, “Prostatic Neoplasms”, “Urinary Bladder Neoplasms”, “Ureteral Neoplasms”, “Cryptorchidism”, “Seminal Vesicles”, “Premature Ejaculation”, “Retrograde Ejaculation”, “Lower Urinary Tract Symptoms”, “Spermatic Cord Torsion”, “Urinary Retention”, “Urinary Catheters”, “Urinary Bladder Neck Obstruction”, “Urinary Bladder Overactive”, “Andrology”, “Infertility Male”, “Urothelium”, “Pudendal Nerve”, “Urination”, “Wolffian Ducts”, “Polycystic Kidney Autosomal Recessive”, “Polycystic Kidney Autosomal Dominant”, “Polycystic Kidney Diseases”, “Polycystic kidneys severe infantile with tuberous sclerosis”, “Medullary Sponge Kidney”, “Wilms Tumor”, “Phimosis”, “Nephrostomy Percutaneous”, “Seminiferous Tubules”.

Two reviewers (J.Y., D.A.) independently reviewed all articles and records that were chosen for analysis. After articles were identified, articles whose primary subject matter did not revolve around urology but rather merely included urology-related terminology were excluded. Any dispute was resolved by discussion with 3rd reviewer (AR). We categorized each article according to the following criteria: (1) article title (2), year of publication (3), country of publication of main author (4), single country of authors (Yes/No) (5), institutional affiliation of corresponding author (6), number of authors (7), type of research (8), publishing house (9), journal name (9), journal impact factor (IF) (11), published date (12), retracted date (13), citations (14), urologic subspecialty, and (15) reason for retraction. We used a cutoff of 10 and 3 for citation count and IF respectively, as per Journal Citation Reports (JCR) for subgroup analysis (DOI:10.13140/RG.2.2.34797.60640).

### Statistical analysis

Continuous variables, like number of citations and time to recall, were represented as means with standard deviations. Wilcoxon tests were conducted to compare the number of citations and time to recall between the two groups. Categorical variables, such as country of publication and article subspecialty, were represented as frequencies and proportions. Additionally, we examined the number of citations in relation to the impact factor using a linear regression model. Statistical analysis was conducted using SAS v9.4 and R version 4.4.1. A p-value < 0.05 was considered statistically significant.

## Results

**Fig. 1:**
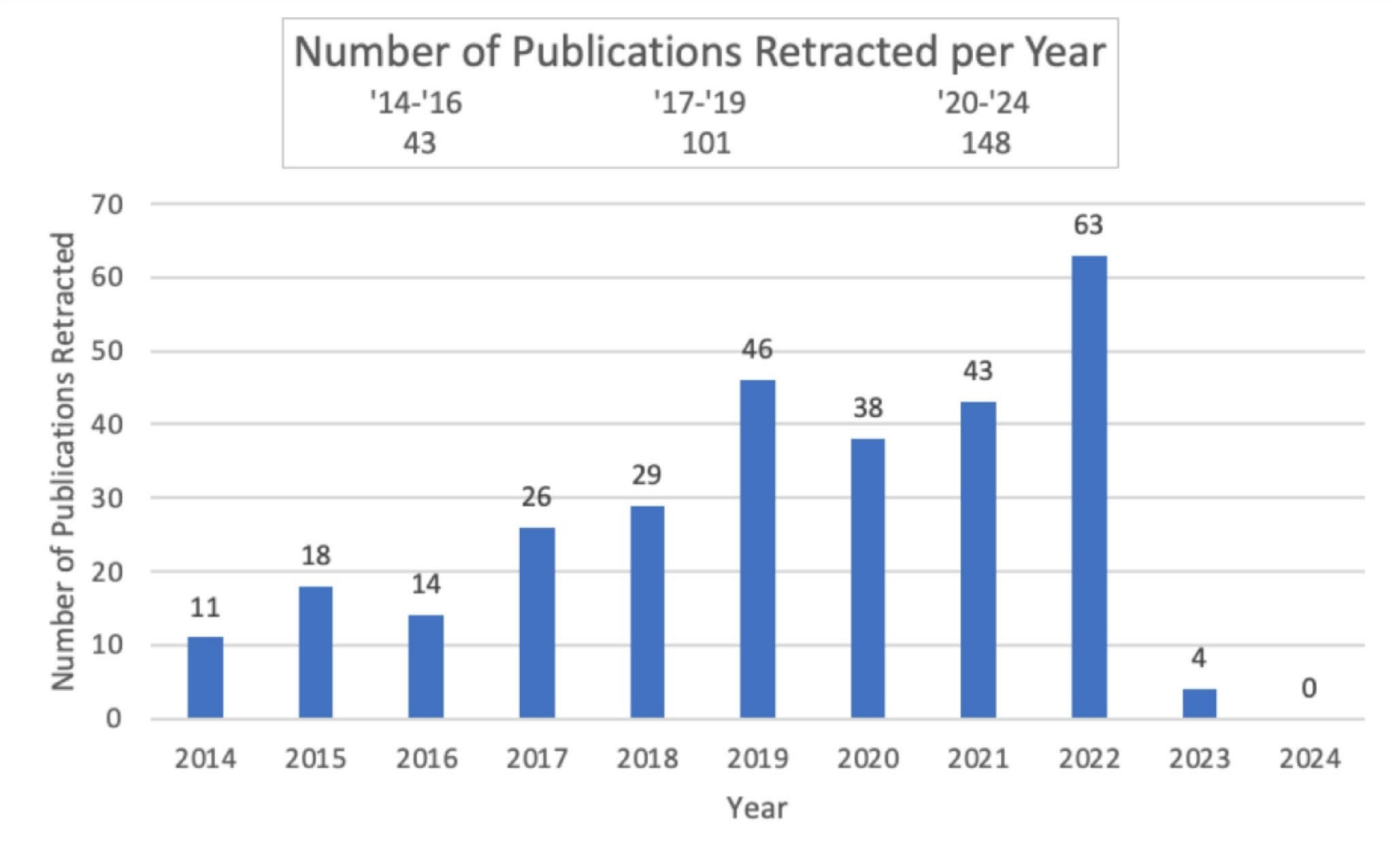
Retraction trends

**Fig. 2:**
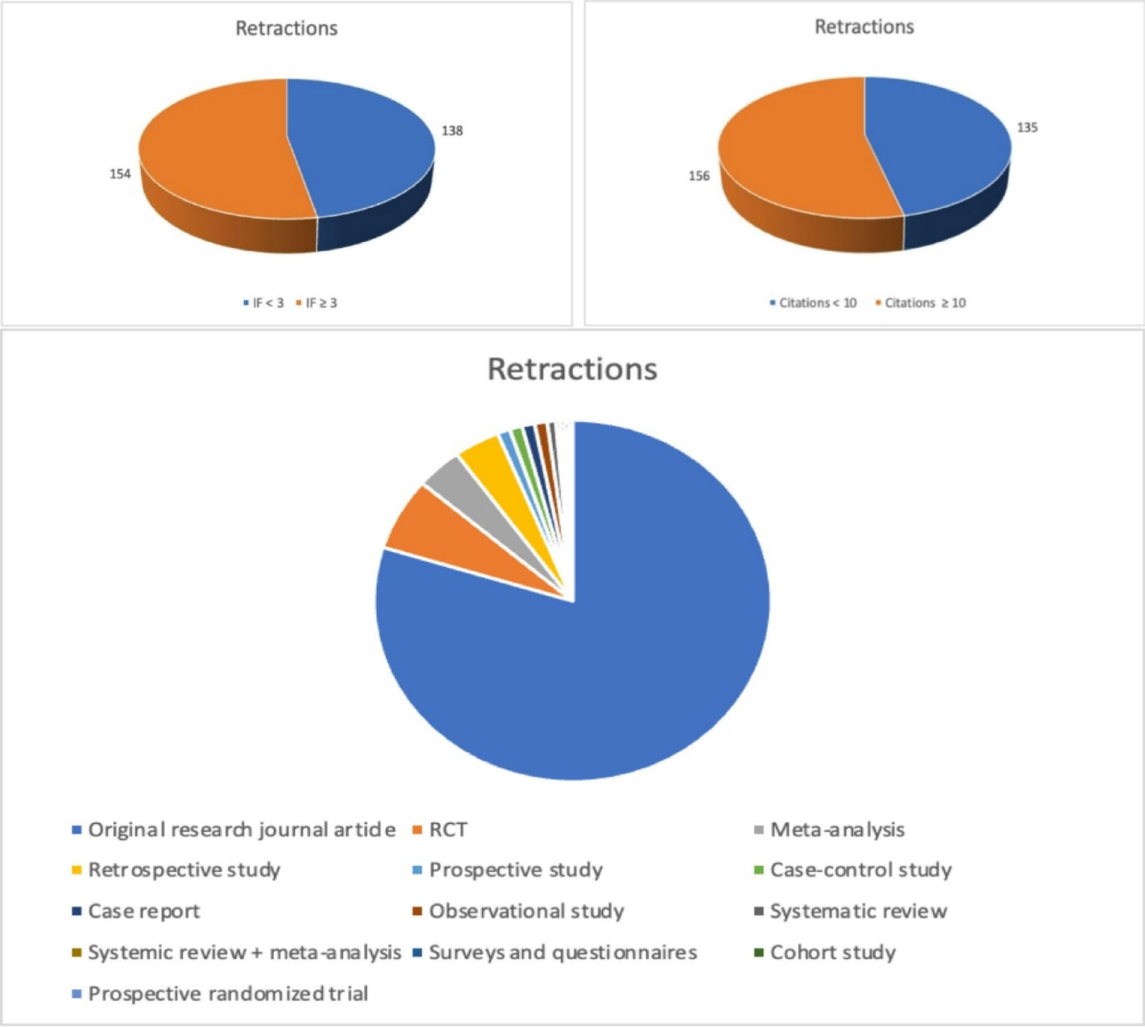
Retractions in regard to impact factor, citation count, and article type

We identified 292 retractions out of 679,874 total papers (0.043%) based on a PubMed keyword search from 2014 to 2024. The number of retractions rose from 43 out of 185,167 between 2014 and 2016 (0.023%) to 101 out of 197,165 between 2017 and 2019 (0.051%) (Fig. 1). Further, between 2020 and 2024 there have been 148 out of 395,259 (0.037%) till now. Most retracted papers were from China (*N* = 247, 84.5%), followed by the United States (*N* = 12, 4.1%) and Iran (*N* = 6, 2.1%). The remaining 27 papers came from 27 different countries. (Table 1). Among the types of articles, 79.7% were original research articles, 3.7% were meta-analyses and retrospective studies each, and the rest were distributed among prospective, case-control, cohort, and observational studies as well as systematic reviews and surveys & questionnaires (Fig. 2).

**Table 1:**
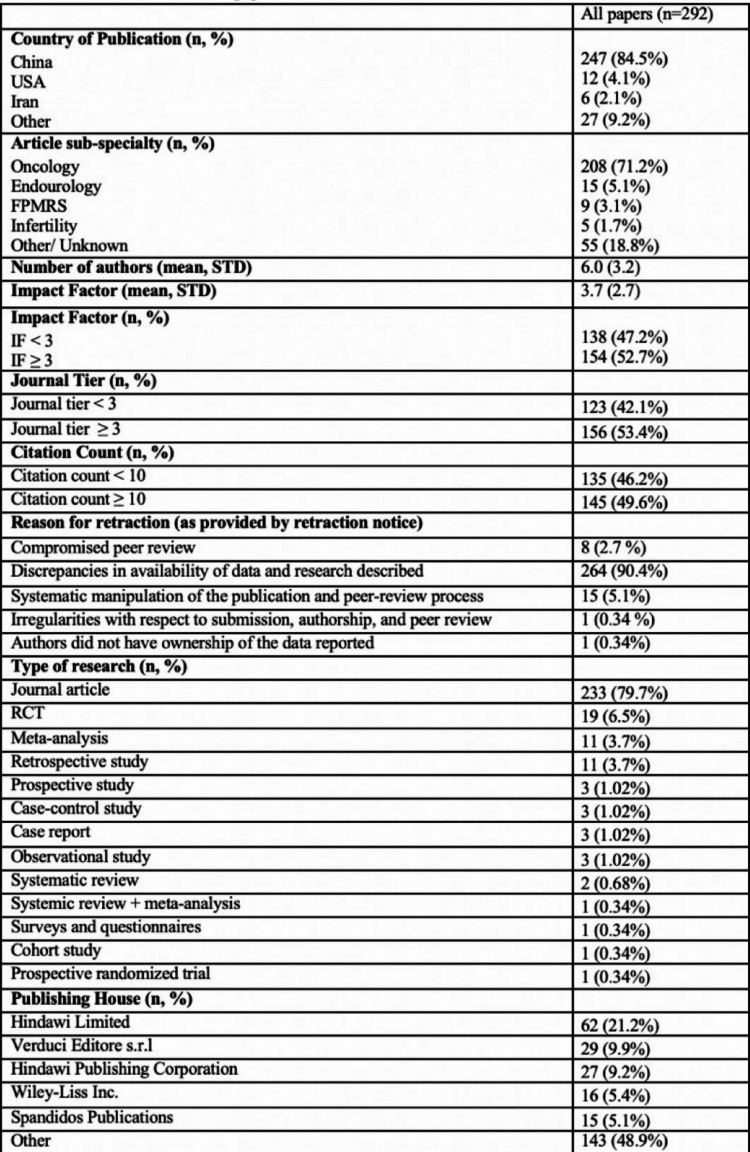
Details of Retracted papers from 2014-2014

Retraction in uro-oncology (*N* = 208, 71.2%) was high, followed by endourology (*N* = 15, 5.1%), Female Pelvic Medicine and Reconstructive Surgery (*N* = 9, 3.1%), infertility (*N* = 5, 7.1%), and other urological fields (*N* = 55, 18.8%). Regarding publishing houses, of the 292 retracted articles, 149 (51.03%) were published by five publishers—Hindawi Limited (62), Verduci Editore (29), Hindawi Publishing Corporation (27), Wiley-Liss (16), and Spandidos Publications (15%). Based off retraction notices, most retractions were mainly due to discrepancies in availability of data and research described (*N* = 264, 90.4%). Other reasons were systematic manipulation of the publication and peer-review process (*N* = 15, 5.1%), compromised peer review (*N* = 8, 2.7%), irregularities with respect to submission, authorship, and peer review (*N* = 1, 0.34%), and authors not having ownership of the data reported (*N* = 1, 0.34%). Among the 292 retracted articles analyzed, 74 (25.3%) were published in subscription-based journals, while 218 (74.7%) appeared in open-access journals (Fig. 2; Table 1).

**Fig. 3:**
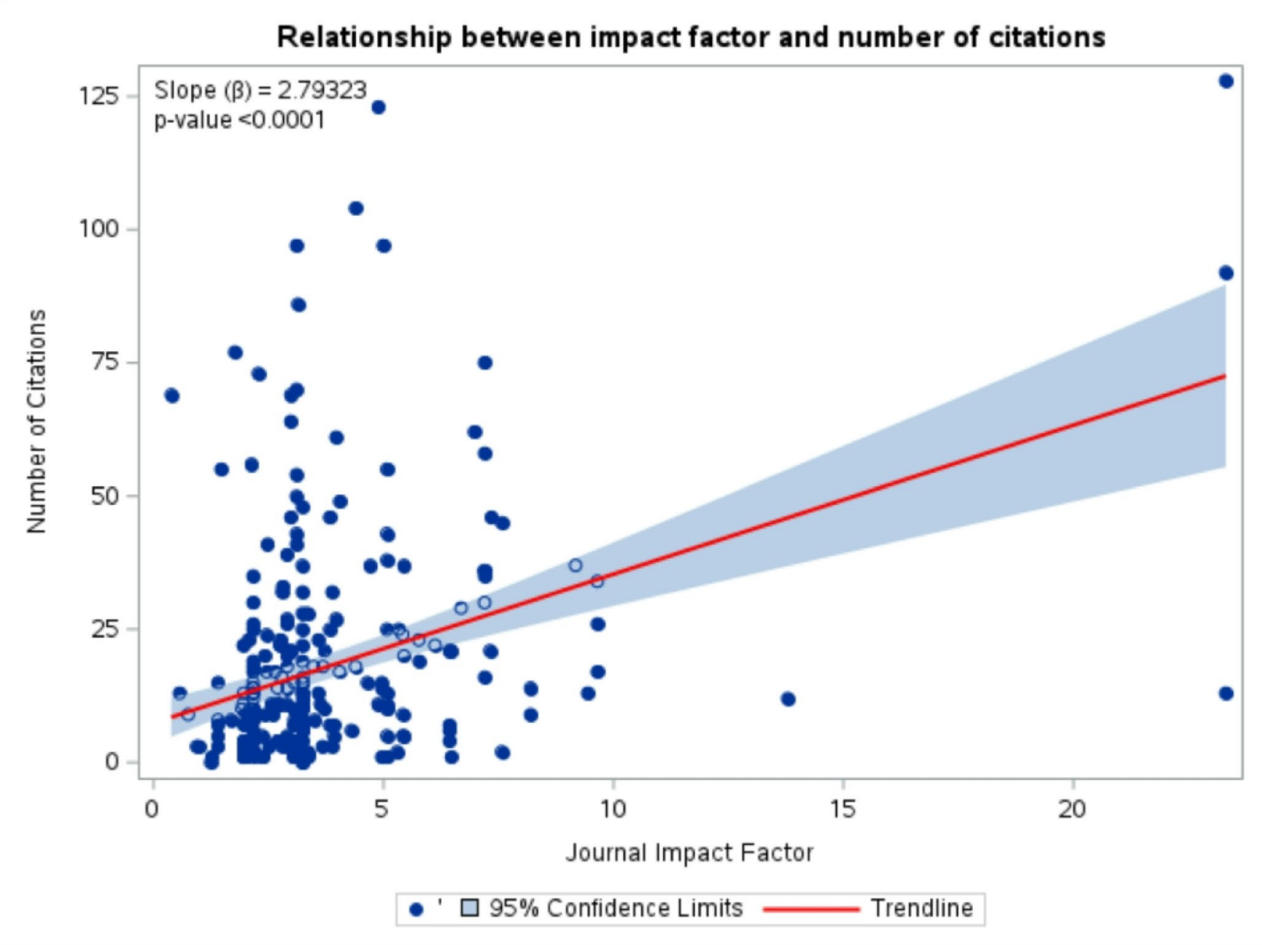
Relationship between impact factor and number of citations

The mean number of authors in the retracted article was 6 *±* 3.2. The mean IF of the retracted articles was 3.7 *±* 2.7. 156 papers (53.4%) had an IF of ≥ 3 (N = and 123 (42.1%) had an IF of < 3. 156 (53.6%) papers had a citation count of ≥ 10 and 135 (41.2%) had a citation count of < 10 (Fig. 2). The mean recall time for journals with an IF > 3 was 35.5 months, compared to 29 months for those with an IF < 3. (Fig. 2). A linear regression model was run to examine the relationship between impact factor and citation count, and the results showed a significant relationship (*p* < 0.0001), meaning the impact factor did influence citation counts. Specifically, for every 1-unit increase in impact factor, the number of citations was expected to increase by 2.79 (Fig. 3).

## Discussion

Our study provides a comprehensive evaluation of retraction trends in urology. Our findings reveal that retractions in urology have steadily increased over the past decade, mirroring trends observed in other medical fields (Fig. 1). This trend aligns with a report by Mena et al., which found that 83% of urology-related retractions from 1999 to 2018 occurred after 2010 [6]. While the overall retraction rate in urology remains lower (4.9 per 10,000 publications) than in orthopedics (8.6) and obstetrics and gynecology (9.1), the upward trend signals the need for enhanced oversight mechanisms [7].

Discrepancies in data availability and research descriptions, including missing data, inconsistencies, or unverifiable findings, emerged as the leading causes of retractions in our study, accounting for 90.4% (*n* = 264). These findings align with those of the Mena et al. study, which identified misconduct such as plagiarism, fake peer review, and data errors as significant contributors to urological retractions, alongside falsification, duplicate publication, and lack of ethical approval [6].

Our study showing original research as the most frequently retracted article type (Fig. 2) is similar to those across various disciplines. For example, Mena et al. (2019) reported that 51% of retracted articles in urology were basic science studies, while Yang et al. (2024) found that 94.17% of retracted biomedical papers were original research [6, 8].

Geographical disparities in research retractions highlight variations in research practices and pressures worldwide. Our study found high retractions from China followed by the United States. This is in contrast to the findings of Mena et al., which indicated a higher rate of retraction in the USA (*n* = 45) compared to China (*n* = 41) between 1999 and 2018 [6]. However, our findings align with research by Shi et al. (2024) on retractions in China, which documented a dramatic increase from 325 in 2018 to 5668 in 2023, primarily in health sciences (21.46%) [9]. This surge parallels the rapid expansion of scientific publications in China [9]. Notably, Chinese authors contribute to over 50% of the papers published in 64 different journals, underscoring the country’s substantial research output [8, 9]. These discrepancies suggest that institutional policies, national research incentives, and regulatory oversight could influence retraction rates across different countries. These findings also align with large-scale analyses of surgical research output that show significant global variation in productivity, often reflecting underlying disparities in human capital and institutional support [10, 11,12]. Web scraping techniques have emerged as a novel method to estimate international collaboration and authorship patterns across disciplines, which could further illuminate factors underlying regional retraction trends and collaboration gaps in urological research [11,13]. Incorporating these digital tools may also help track evolving networks of scientific collaboration and identify vulnerabilities in editorial oversight.

Our study found that high-impact journals take more time to retract than low-impact journals. This aligns with Mena et al., who reported a median recall time of 30 months for retractions in urology journals, suggesting that high-impact journals may conduct more thorough investigations before retracting a paper [6]. However, despite this increased oversight, there was no significant difference in citation counts at the time of retraction (*p* = 0.062), indicating that flawed research continues to be cited at similar rates regardless of a journal’s impact factor.

Our study also found a predominance of retracted articles in open-access journals similar to Tripathi et al. (2019), who reported that open-access journals account for 73.8% of total retractions and have a retraction rate nearly three times higher than that of subscription-based journals in fields such as oncology, biochemistry and molecular biology, and research and experimental medicine [14]. This trend may be driven by financial pressures and varying peer-review standards, though open-access journals differ widely in quality [14]. While some maintain rigorous oversight, others may be more susceptible to lapses, contributing to higher retraction rates [14]. However, research by Björk et al. (2012) suggests that open-access journals, particularly those funded by article processing charges, can achieve high scientific impact [15]. The assumption that open-access journals inherently have lower standards is flawed, as citation disparities largely disappear when accounting for discipline, journal age, and publisher location [14, 15].

Our study found that uro-oncology accounted for 71.2% of all retractions, similar to Mena et al. who reported 70% of urological retractions were related to oncology research. This reinforces the idea that subspecialties with a high volume of publications may be more susceptible to retraction due to complex data interpretation and the competitive nature of publishing [6].

While our study identified multiple statistically significant associations—such as between impact factor and citation count or retraction frequency and geographic origin—these findings do not imply causality, but rather reflect a correlational effect. As Karamitros et al. emphasize in their recent guide on causal inference in clinical research, observational analyses can be misleading if interpreted through a causal lens without appropriate methodology such as instrumental variable analysis or longitudinal modeling [12]. Future research using causal inference frameworks could better assess whether specific publishing models or editorial policies actively influence retraction likelihood.

Our research has several limitations. First, while our search strategy was thorough, it was limited to PubMed and relied on MeSH terms. This may have resulted in excluding retracted articles indexed in other databases, such as Scopus or Web of Science. Additionally, the format and level of detail in retraction notices varied among different publishers, which required us to classify the reasons for retraction. Although this facilitated comparisons, it might have led to the underrepresentation of specific types of misconduct. Lastly, while we assessed citation counts at the time of retraction, we did not differentiate between positive and negative citations. This omission may affect the interpretation of citation impact following retraction. Furthermore, our exclusion of non-PubMed indexed literature may skew geographic distribution and representation of publishers, particularly for journals that are indexed locally or regionally. Future studies could integrate Scopus, Web of Science, or CrossRef databases to provide a more complete assessment [15].

## Conclusion

This study examines the rising trend of retractions in urological research, especially in high-impact areas like oncology, where misconduct and methodological flaws are common. Factors such as geographic disparities, open-access models, and journal impact significantly influence retraction patterns. To strengthen research integrity, standardized reporting guidelines, mandatory data transparency, and better institutional oversight are essential. As the landscape of academic publishing evolves with issues like paper mills and predatory journals, ongoing research is needed to monitor these trends and their impact on scientific credibility and patient care.

## Data Availability

No datasets were generated or analysed during the current study.
